# Seasonal regulation of selenium biofortification in tea through Se-rich organic fertilizer application

**DOI:** 10.3389/fpls.2026.1883624

**Published:** 2026-07-15

**Authors:** Qing Liao, Jin-Ping Chen, Ying Xing, Jing-Xin Huang, Yong-Xian Liu, Li-Ping Pan, Qin Li, Jin-Huan Qin, Cheng-Shu Zhou, Lin Xie, Zhu-Sheng Liu

**Affiliations:** 1 Agricultural Resources and Environmental Research Institute, Guangxi Academy of Agricultural Sciences/Guangxi Key Laboratory of Arable Land Conservation, Nanning, China; 2 Beiyi Tea Factory, Guigang, China; 3 Guangxi Tea Research Institute, Guilin, China

**Keywords:** antioxidant enzyme, *Camellia sinensis*, organic matter (OM), selenium (Se), soil enzyme, tea quality

## Abstract

Selenium (Se) deficiency affects approximately one billion people globally, and Se biofortification of tea represents a promising strategy to enhance dietary Se intake while improving tea quality; however, the seasonal dynamics and dose-dependent effects of Se-enriched organic fertilizers in perennial tea systems remain poorly understood. This study evaluated the impacts of three Se-enriched organic fertilizer application rates on soil properties, Se accumulation, enzymatic activities, and tea quality across spring, summer, and autumn harvests in a subtropical tea plantation in Guangxi, China. Soil available Se followed a consistent dose hierarchy (T3 > T2 > T1 > CK) and declined progressively across seasons, yet T3 demonstrated superior retention, declining only 29.1% from spring to autumn versus 53.3% for CK, sustaining autumn soil Se at 0.0559 mg kg^-1^ (+175.4% over CK). Leaf Se content increased 2.04-3.54-fold over CK across seasons, with T2 achieving the greatest autumn enhancement (0.6660 mg kg^-1^). Soil enzyme responses were highly season-specific: summer urease activity under T2 collapsed by 55.3% relative to CK before reversing to a +174.7% peak in autumn, while summer invertase was dose-dependently suppressed (up to -45.8% under T3) and acid phosphatase was uniformly elevated (up to +34.1%). In tea leaves, high-dose Se (T2/T3) elevated spring GSH-Px activity ~94% above CK, while autumn CAT activity increased 7.3-fold under T2. Se treatments protected summer chlorophyll from stress-induced degradation, raising total chlorophyll by up to 18.8%. Regarding tea quality, T3 most effectively improved summer tea by reducing the polyphenol-to-amino acid (P/A) ratio by 22.6% and increasing free amino acids by 20.9%, while T2 was optimal for autumn tea, maximizing water extractive content at 37.53% (+12.6%) and free amino acids at 3.03% (+9.8%). These findings establish season-specific optimal application rates for simultaneous dietary Se enrichment and tea quality improvement, providing an evidence base for commercial Se biofortification recommendations.

## 1 Introduction

Tea (*Camellia sinensis* L.), one of the most widely consumed non-alcoholic beverages worldwide, is cultivated on over 4.9 million hectares across more than 60 countries and represents a primary daily source of bioactive compounds, including polyphenols, amino acids, and minerals, for billions of consumers globally (Zhao et al., 2022; Luo et al., 2024). Beyond its cultural and economic significance, tea has attracted sustained scientific interest as a functional food whose phytochemical profile is highly responsive to agronomic management, soil chemistry, and environmental conditions (Yang et al., 2025; Ye et al., 2023; Ahmed et al., 2019). Among the mineral elements influencing both tea plant physiology and human health, selenium (Se) has emerged as a micronutrient of exceptional importance, situated at the intersection of soil biogeochemistry, plant nutrition, and preventive medicine.

Selenium is an essential trace element for humans and animals, functioning as a catalytic component of selenoproteins, including glutathione peroxidases (GSH-Px), thioredoxin reductases, and selenoprotein P, that are indispensable for antioxidant defense, thyroid hormone metabolism, immune function, and the prevention of oxidative stress-associated diseases such as cardiovascular disease, certain cancers, and neurodegenerative disorders (Rotruck et al., 1973; Rayman, 2012; Liang et al., 2023; Bai et al., 2024; Li et al., 2025; Qadir and Omer, 2026). The recommended dietary intake of Se for adults ranges from 60 to 400 μg day^-1^ (Zhao et al., 2018); yet an estimated one billion people worldwide remain Se-deficient, predominantly in regions where soils are inherently low in Se or where Se bioavailability is compromised by acidic pH, or competitive ion interactions (Haug et al., 2007; Qin et al., 2012; Su and Suarez, 2000). China, a country characterized by extensive seleniferous and Se-deficient soil zones distributed along a characteristic geological belt, presents a complex landscape in which large rural and agricultural populations face chronic inadequate Se intake (Li et al., 2017; Yang et al., 2022).

Tea gardens in Guangxi, China are preferentially established on acidic, weathered red soils in subtropical and tropical regions, precisely the soil types characterized by low Se bioavailability due to strong adsorption of selenite by iron and aluminum oxyhydroxides under acidic conditions (Liao et al., 2024a; Zhao et al., 2024). The strongly acidic pH typical of tea garden soils (commonly pH 4.0-5.5) not only reduces Se solubility but also exacerbates aluminum toxicity and suppresses beneficial soil microbial activity, creating a multi-stressor environment that simultaneously constrains nutrient cycling and plant nutrient uptake (Zhang et al., 2006; Yan et al., 2018; Zheng et al., 2022; Yang et al., 2023; Jiang et al., 2024). Given that tea is consumed in large quantities and that the Se concentration of tea leaves is directly determined by soil Se availability and plant uptake efficiency, selenium biofortification of tea, elevating leaf Se to physiologically meaningful concentrations without impairing quality attributes, represents an attractive and scalable dietary intervention strategy (Sarwar et al., 2020; Xiang et al., 2022; An et al., 2025).

Previous studies have demonstrated that Se can be applied to tea plants via foliar spraying, soil amendment, or irrigation, with soil application offering more sustained enrichment due to reservoir effects in the rhizosphere (Li et al., 2021; Xiang et al., 2022; An et al., 2025). Among the Se forms used in soil application, selenite and selenate have been most extensively studied; however, both are subject to rapid immobilization, leaching, or volatilization depending on soil redox status and microbial activity (Liao et al., 2024a). The integration of Se into organic fertilizers offers a theoretically superior delivery system, organic matter can serve as a slow-release Se carrier, simultaneously ameliorating soil acidification, enhancing microbial biomass and enzymatic activity, and improving soil structure, factors that collectively favoring Se bioavailability and plant uptake (Gui et al., 2022; Jiang et al., 2021; Li et al., 2021; Yang et al., 2022; Zhi et al., 2023). Se-enriched organic fertilizers, in which Se is chelated or complexed within organic matrices derived from Se-accumulating plant biomass or Se-enriched microbial biomass, represent a relatively novel category of agronomic input whose effects on soil-plant Se cycling in perennial plantation crops remain incompletely characterized.

Soil enzyme activities, including catalase, urease, invertase, and acid phosphatase, are widely recognized as sensitive bioindicators of soil biochemical functioning, microbial community composition, and nutrient cycling efficiency (Martín-Sanz et al., 2018; Galhardi et al., 2020). Their responses to Se amendment are, however, highly context-dependent: Se at low concentrations may stimulate enzymatic activity by promoting microbial growth and alleviating oxidative stress, whereas excess Se can be inhibitory through direct protein denaturation or disruption of microbial community structure (Martín-Sanz et al., 2018; Liao et al., 2024a). Critically, the seasonal context, encompassing temperature, moisture, plant growth stage, and rhizosphere exudate composition, modulates Se speciation, soil microbial dynamics, and enzyme kinetics in ways that remain poorly understood, particularly in multi-harvest perennial systems such as tea plantations, where three distinct flush seasons (spring, summer, and autumn) impose dramatically different physiological and environmental conditions on both soil and plant.

The quality of tea is governed by a complex interplay of primary metabolites, principally polyphenols (catechins), free amino acids (theanine), chlorophylls, and water-soluble extractives, whose relative concentrations determine sensory attributes including astringency, umami flavor, color, and overall liquor richness (Borowik et al., 2017; Cheng et al., 2024). The polyphenol-to-amino acid (PA) ratio is particularly valued as a composite quality index, with lower values associated with superior flavor profiles in green tea (Guo et al., 2026). Selenium has been reported to influence the biosynthesis and accumulation of these metabolites through its roles in antioxidant enzyme activation, reactive oxygen species (ROS) scavenging, and modulation of carbon and nitrogen metabolism (Fang et al., 2026; Guo et al., 2026). However, the extent to which such effects are dose- and season-specific in a three-harvest-per-year production system has not been systematically elucidated.

Despite growing interest in Se biofortification of tea, season-specific dose optima for Se-enriched organic fertilizers remain undefined. Addressing these gaps is essential for designing Se biofortification programs that maximize health benefits and tea quality while avoiding phytotoxicity or ecological risk. The present study therefore investigated the effects of three Se-enriched organic fertilizer application rates on soil and plant Se dynamics, soil enzyme activities, plant antioxidant responses, and biochemical quality indicators across spring, summer, and autumn harvests in a representative Guangxi tea plantation. This work aimed to characterize the seasonal dynamics of Se fate in the soil-plant system, elucidate enzyme- and season-specific effects on soil biochemical functioning; determine dose-response relationships governing Se accumulation and quality enhancement; and identify season-specific optimal application rates for the simultaneous improvement of dietary Se content and tea sensory quality, thereby providing an evidence base for commercial Se biofortification recommendations.

## 2 Material and methods

### 2.1 Materials

The tea cultivar used in the trial was YingShuang, 13 years old. The selenium fertilizer applied was a selenium-enriched organic fertilizer supplied by Suzhou Xigu Technology Co., Ltd., with NPK ≥ 4%, organic matter ≥ 30%, pH 6.43, and selenium content 1000 mg/kg. The trial site was located at the Beiyi Tea Factory plantation base, Gangnan District, Guigang City, Guangxi (109°39′2″E, 22°48′38″N; elevation 129.3 m). The soil was red loam; its physicochemical properties are shown in **Table 1**.

**Table 1:** Physicochemical properties and selenium content of the soil.

Total Nitrogen (g/kg)	Total phosphorus (g/kg)	Total potassium (g/kg)	Available nitrogen (mg/kg)	Available phosphorus (mg/kg)	Available potassium (mg/kg)	pH value	Organic matter (g/kg)	Total Se content (mg/kg)
3.37	2.38	20.56	238.5	227.0	81.7	3.96	49.1	0.75

### 2.2 Experimental design and sampling

Four fertilizer treatments were established according to application rate: 0 (CK), 150 (T1), 225 (T2) and 300 kg ha^−1^ (T3). Fertilizer was applied in trenches dug 15-20 cm from the root zone on the inner side of each tea row and covering with a thin layer of soil. The experimental plot used a terraced, double-row planting system; plot length was 10 m, row spacing 1.8 m, three rows per plot, and each treatment was replicated three times. All other agronomic management followed standard local tea-crop practices.

Tea and soil samples were collected at three harvests: spring (17 April 2025), summer (17 July 2025) and autumn (16 October 2025). The meteorological information for these months at experiment site is showed in **Supplementary Table 1**. For each sample, shoots consisting of the top two leaves plus the bud were excised, rinsed three times with distilled water, blotted dry on filter paper and processed as follows: fresh material was used immediately to determine antioxidant enzyme activities and chlorophyll content; the remaining material was fixed at 105 °C for 5 min, oven-dried at 55 °C to constant weight, then ground and sieved for subsequent chemical analyses. Rhizosphere soil was collected from the root zone, air-dried, ground, and sieved. The fractions retained/passed through 2 mm, 30-mesh, 60-mesh and 100-mesh sieves were used for soil pH measurement, soil enzyme-activity assays, determination of available selenium and selenium speciation analysis, respectively.

### 2.3 Measurement of soil samples

The activities of soil catalase, soil invertase, soil sucrase, soil acid phosphatase, and soil available selenium content were measured according to Liao et al. (2025).

Soil pH was determined by the potentiometric method following the Chinese standard HJ 962-2018 (Determination of soil pH by potentiometry). Soil was mixed with deionized water at a soil:water ratio of 1:2.5 (w/v), equilibrated (gentle shaking) for 30 min, and pH was measured with a calibrated pH meter at room temperature. Measurements were made in triplicate for each sample; the meter was calibrated with standard buffers (pH 4.00, 7.00 and 10.00) before use and checked periodically during runs.

### 2.4 Measurement of plant samples

Plant glutathione peroxidase (GSH-Px) activity was determined using a commercial activity assay kit (Beijing Solarbio Science & Technology Co., Ltd., Beijing, China) according to the manufacturer’s protocol. Superoxide dismutase (SOD), catalase (CAT), and peroxidase (POD) activities were measured using commercial assay kits (Suzhou Keming Biotechnology Co., Ltd., Suzhou, China) following the manufacturer’s instructions. Enzyme activities were expressed as follows: GSH-Px, U g^-1^ fresh weight (FW); SOD, U g^-1^ FW; CAT, μmol min^-1^ g^-1^ FW; POD, U g^-1^ FW.

Free amino acid content was determined by the ninhydrin colorimetric method according to the Chinese National Standard GB/T 8314-2013 (Standardization Administration of China, 2013). Briefly, free amino acids in tea extracts reacted with ninhydrin under weakly acidic conditions (pH 8.0 phosphate buffer) at 100 °C for 15 min to form a purple chromophore, which was measured spectrophotometrically at 570 nm. A standard calibration curve was constructed using L-theanine as the reference standard, and results were expressed as mg g^−^¹ dry weight (DW) equivalent to theanine.

Chlorophyll content, polyphenol content, and plant selenium content were determined according to the methods described by Liao et al. (2025).

### 2.5 Data analysis

Statistical analyses were conducted using Microsoft Excel 2017 and SPSS version 19.0. A one-way ANOVA was performed, followed by Duncan’s multiple range test for pairwise comparisons. Experiments were conducted in triplicate, and results were presented as mean ± standard error. Significance was defined as p < 0.05 (significant).

## 3 Results

### 3.1 Regulatory effects of various treatments on soil pH in tea plantations across seasons

Soil pH values ranged from 3.83 to 4.15 across all treatments and seasons, consistently reflecting the strongly acidic nature of the tea garden soil (**Table 2**). Significant treatment effects on soil pH were observed in spring and summer, but not in autumn.

**Table 2:** Effect of various treatments on soil pH.

Treatment	Spring	Summer	Autumn
CK	3.93 ± 0.06ab	3.83 ± 0.04b	3.88 ± 0.05a
T1	3.84 ± 0.01b	4.04 ± 0.01a	3.86 ± 0.01a
T2	4.05 ± 0.02a	4.03 ± 0.01a	4.15 ± 0.01a
T3	3.89 ± 0.04b	3.97 ± 0.01a	4.07 ± 0.01a

Different lowercase letters within the same column indicate significant differences among treatments in the same season (P<0.05).

In spring, T2 treatment produced the highest pH value across all treatments and seasons (4.05 ± 0.02), significantly exceeding CK (3.93 ± 0.06), T1 (3.84 ± 0.01), and T3 (3.89 ± 0.04) (p < 0.05), while T1 and T3 did not differ significantly from CK. In summer, T1, T2, and T3 all significantly elevated soil pH relative to CK (3.83 ± 0.04), yielding comparable values of 4.04 ± 0.01, 4.03 ± 0.01, and 3.97 ± 0.01, respectively, with no significant differences among the three selenium treatments, a pattern indicative of a threshold effect in which even the lowest selenium dose achieved the maximum attainable pH increase. In autumn, soil pH showed an increasing trend from CK (3.88 ± 0.05) through T1 (3.86 ± 0.01), T3 (4.07 ± 0.01), and T2 (4.15 ± 0.01); however, none of these differences reached statistical significance. Overall, selenium application demonstrated the most pronounced and treatment-differentiated effect on soil pH in spring, whereas summer pH responses showed uniform elevation across all selenium doses.

### 3.2 Seasonal dynamic regulatory effects of various treatments on soil enzyme activities in tea plantations.

**Figure 1:**
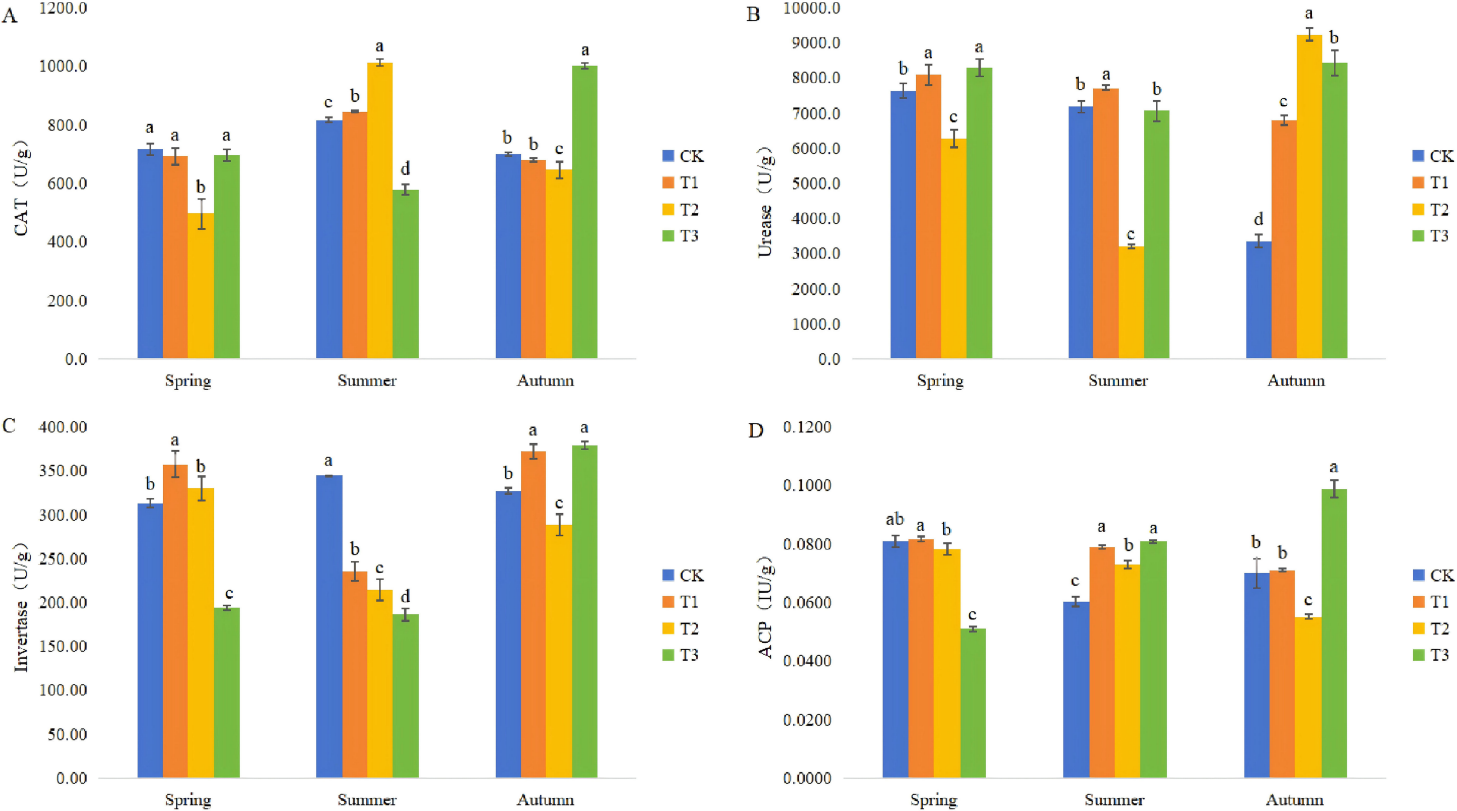
Effect of various treatments on soil enzyme activities. **(A)** catalase; **(B)** urease; **(C)** invertase; **(D)** acid phosphatase. In each season, different lowercase letters denote significant differences among treatments (P < 0.05).

Soil enzyme activities showed significant seasonal and treatment-dependent variations closely associated with meteorological conditions, particularly temperature, precipitation, and humidity (**Figure 1**; **Supplementary Table 2**).

Catalase (CAT) activity peaked in summer under CK, T1, and T2 (818.53, 847.17, and 1013.60 U/g, respectively), coinciding with peak temperature (28.9°C) and humidity (86%), suggesting elevated thermal and moisture conditions stimulated microbial oxidative stress responses. T2 recorded the highest summer CAT activity among all treatments (p < 0.05). T3 showed a contrasting pattern, with minimum CAT activity in summer (579.53 U/g) and a peak in autumn (1003.53 U/g), suggesting T3 buffered oxidative stress during summer while enhancing antioxidant activity under milder autumn conditions (24.5°C, 79% humidity) (**Figure 1A**).

Urease activity exhibited pronounced seasonal dynamics. CK showed the highest spring activity (7649.27 U/g) but declined sharply in autumn (3365.77 U/g, -56%), reflecting limited nitrogen mineralization capacity without amendments. T3 maintained consistently elevated urease levels from spring (8292.23 U/g) through autumn (8445.17 U/g), indicating enhanced nitrogen cycling stability. T2 showed the greatest fluctuation, collapsing in summer (3216.47 U/g), likely due to inhibitory effects of intense precipitation (234.1 mm) on microbial nitrogen transformation, before recovering to its seasonal maximum in autumn (9247.97 U/g) (**Figure 1B**).

Invertase activity was broadly suppressed in summer across most treatments, with T1 and T2 recording their lowest values (235.83 and 214.67 U/g), consistent with excessive soil moisture reducing substrate availability for sucrose hydrolysis. T3 achieved the highest invertase activity across all treatments in autumn (379.67 U/g, p < 0.05), suggesting moderate autumn conditions created optimal circumstances for soil carbon metabolism (**Figure 1C**).

Acid phosphatase (ACP) activity generally declined from spring to summer under CK and T2, reflecting inhibitory effects of high temperature and moisture on phosphorus mineralization. T1 maintained relatively stable ACP activity year-round, suggesting a stabilizing effect on phosphorus cycling. T3 again showed a contrasting response, with the lowest spring ACP activity (0.0511 IU/g) and highest autumn activity (0.0990 IU/g), the latter representing the maximum across all treatments. This autumn peak, concurrent with elevated urease and invertase activities under T3, suggests coordinated enhancement of soil carbon, nitrogen, and phosphorus cycling under cooler, moderately humid autumn conditions (**Figure 1D**).

Overall, seasonal climatic transitions substantially influenced soil enzyme activities. T3 exhibited the most consistent multi-enzyme enhancement in autumn, while T2 demonstrated the greatest seasonal sensitivity to summer thermal and hydrological extremes.

### 3.3 The effects of various treatments on soil available Se content exhibited treatment- and season-dependent dynamics

**Figure 2:**
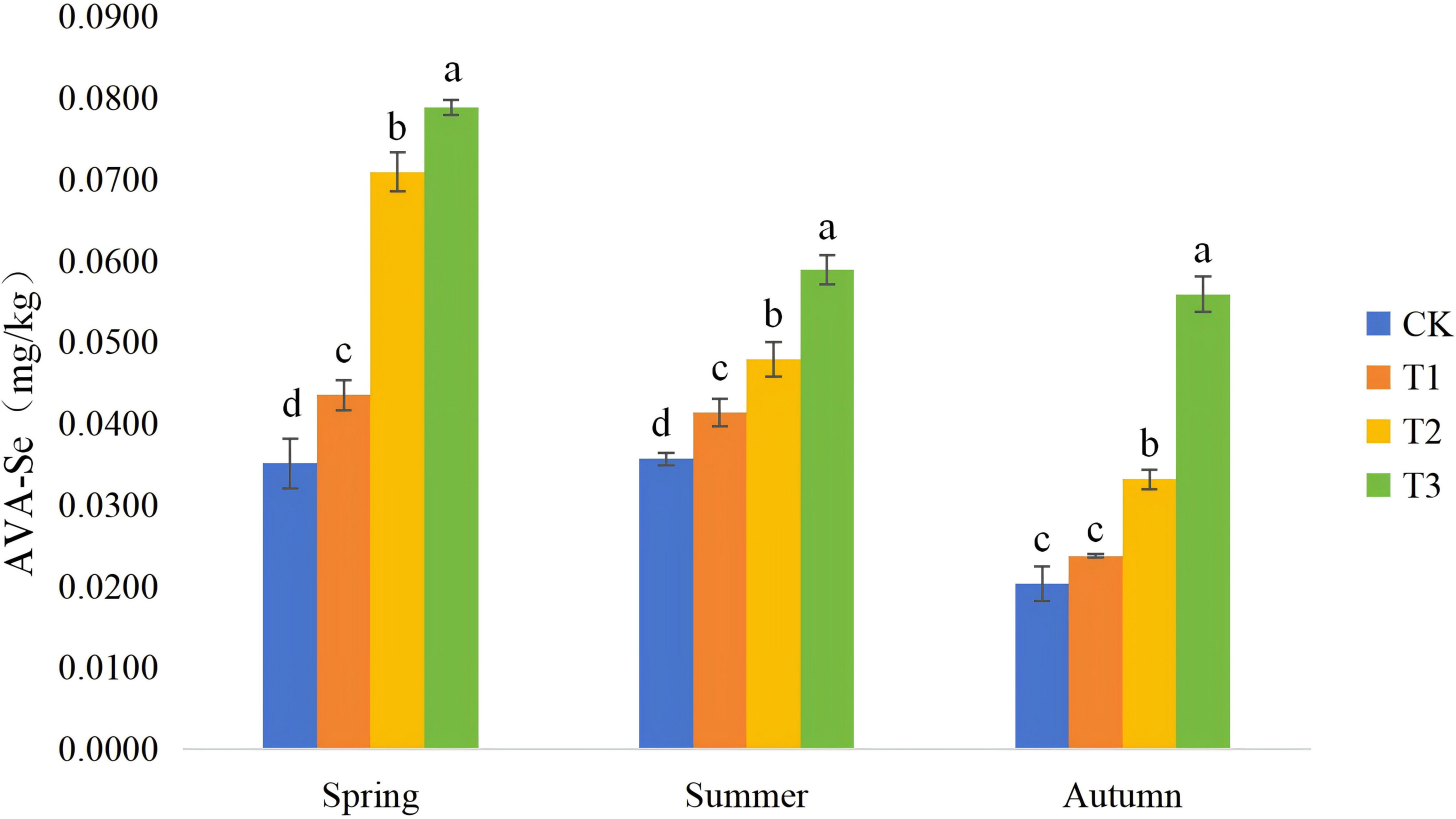
Effect of various treatments on soil available Se content. Different lowercase letters indicate significant differences among treatments in the same season (P<0.05). AVA-Se: available Se.

The soil available selenium (Se) content varied significantly among treatments and across seasons. A consistent treatment hierarchy was observed throughout: T3 > T2 > T1 > CK, indicating that higher exogenous Se application persistently elevated soil available Se regardless of seasonal fluctuations (**Figure 2**; **Supplementary Table 3**).

All treatments showed a progressive decline in soil available Se from spring to autumn, closely associated with seasonal precipitation patterns. Autumn recorded the lowest Se values across all treatments, coinciding with cumulative leaching effects following the high-precipitation summer (234.1 mm), which likely accelerated Se translocation to deeper soil horizons and promoted conversion to less bioavailable fractions. Spring, with minimal precipitation (82.5 mm) and lowest humidity (70%), yielded the highest seasonal Se concentrations, further confirming the role of hydrological conditions in governing Se retention and bioavailability (**Figure 2**).

Treatment-induced Se enrichment relative to CK intensified from spring to autumn. In spring, T2 and T3 exceeded CK by 102.0% and 124.5%, respectively; by autumn, these differences widened to 63.1% and 175.4%. Specially, T3 demonstrated superior Se retention, declining only 29.1% from spring to autumn, compared to 53.3%, 45.5%, and 42.2% for T2, T1, and CK, respectively (**Figure 2**).

T2 exhibited the greatest seasonal sensitivity, recording the largest spring-to-summer Se decline (32.6%), paralleling the concurrent collapse of urease activity under summer climatic extremes, suggesting disproportionate disruption of soil biogeochemical functioning under T2 management (**Figure 2**).

In contrast, T3 maintained the highest available Se across all seasons, with its autumn value (0.0559 mg/kg) remaining markedly elevated relative to all other treatments. This sustained Se availability, coupled with concurrent autumnal peaks in urease, invertase, and acid phosphatase activities, suggests that T3 established the most favorable soil nutritional environment during the moderate autumn conditions (24.5°C, 79% humidity), providing a biochemically supportive foundation for Se uptake by tea plants (**Figure 2**).

### 3.4 Various treatments significantly altered the Se content in tea leaves

**Figure 3:**
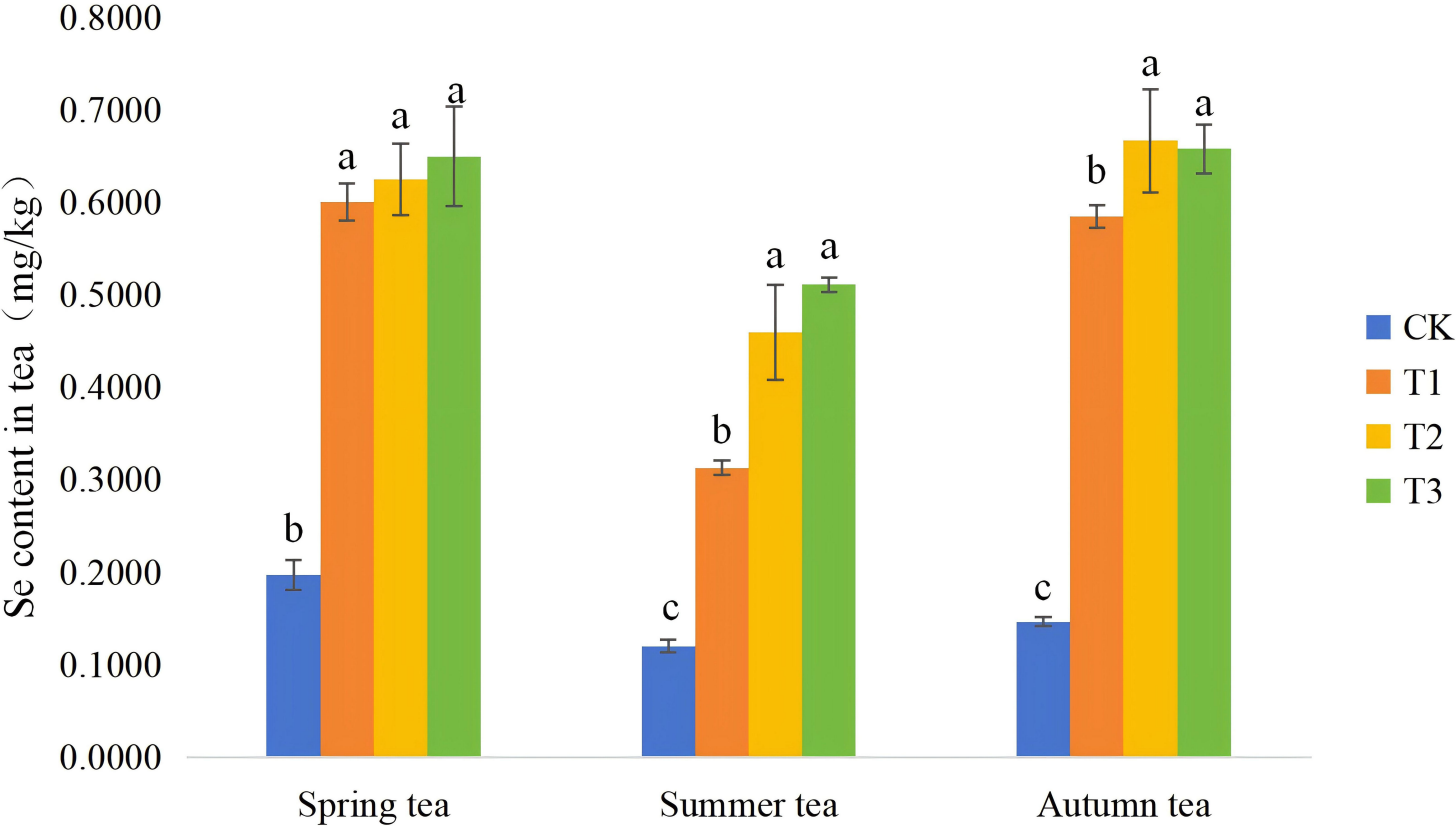
Effect of various treatments on Se content in tea. Different lowercase letters indicate significant differences among treatments in the same season (P<0.05).

Se content in tea leaves was significantly higher in all Se-treated groups (T1-T3) than CK (p < 0.05) and generally increased with application rate. Seasonal patterns differed by dose: CK and T1 followed spring > autumn > summer, while T2 and T3 showed autumn ≥ spring > summer, indicating a unique promoting effect of higher doses on autumn Se accumulation ((**Figure 3**; **Supplementary Table 4**).

In spring, T1-T3 contents (0.5998 ± 0.0204, 0.6240 ± 0.0385, and 0.6491 ± 0.0537 mg kg^-1^) were all significantly higher than CK (0.1970 ± 0.0164 mg kg^-1^; p < 0.05), but did not differ significantly among themselves, suggesting tea plants reached an Se uptake saturation threshold in spring despite increasing soil availability. Increases over CK ranged from 2.04- to 2.29-fold (**Figure 3**).

In summer, Se content fell to its annual minimum with clear dose-dependent differentiation (p < 0.05): T3 (0.5105 ± 0.0079 mg kg^-1^) > T2 (0.4590 ± 0.0510 mg kg^-1^) > T1 (0.3127 ± 0.0079 mg kg^-1^) > CK (0.1199 ± 0.0067 mg kg^-1^), mirroring the soil available Se ranking exactly and indicating that soil Se availability directly limited uptake efficiency. T3 produced the largest seasonal enhancement at +3.26-fold over CK (**Figure 3**).

In autumn, Se content rebounded markedly. T2 (0.6660 ± 0.0554 mg kg^-1^) and T3 (0.6572 ± 0.0262 mg kg^-1^) significantly exceeded T1 (0.5840 ± 0.0123 mg kg^-1^) and CK (0.1465 ± 0.0048 mg kg^-1^). Notably, T2 autumn Se content significantly surpassed its spring value, while T3 showed no significant difference between seasons. Despite T1 soil available Se being statistically indistinguishable from CK in autumn, T1 tea Se remained significantly elevated, suggesting plants may adopt alternative uptake strategies (e.g., deep root absorption) to sustain accumulation. T2 achieved the largest enhancement across all seasons and treatments (+3.54-fold over CK) (**Figure 3**).

### 3.5 The regulation of four key antioxidant enzyme activities in tea by various treatments exhibited enzyme- and season-specific and dose-dependent patterns

**Figure 4:**
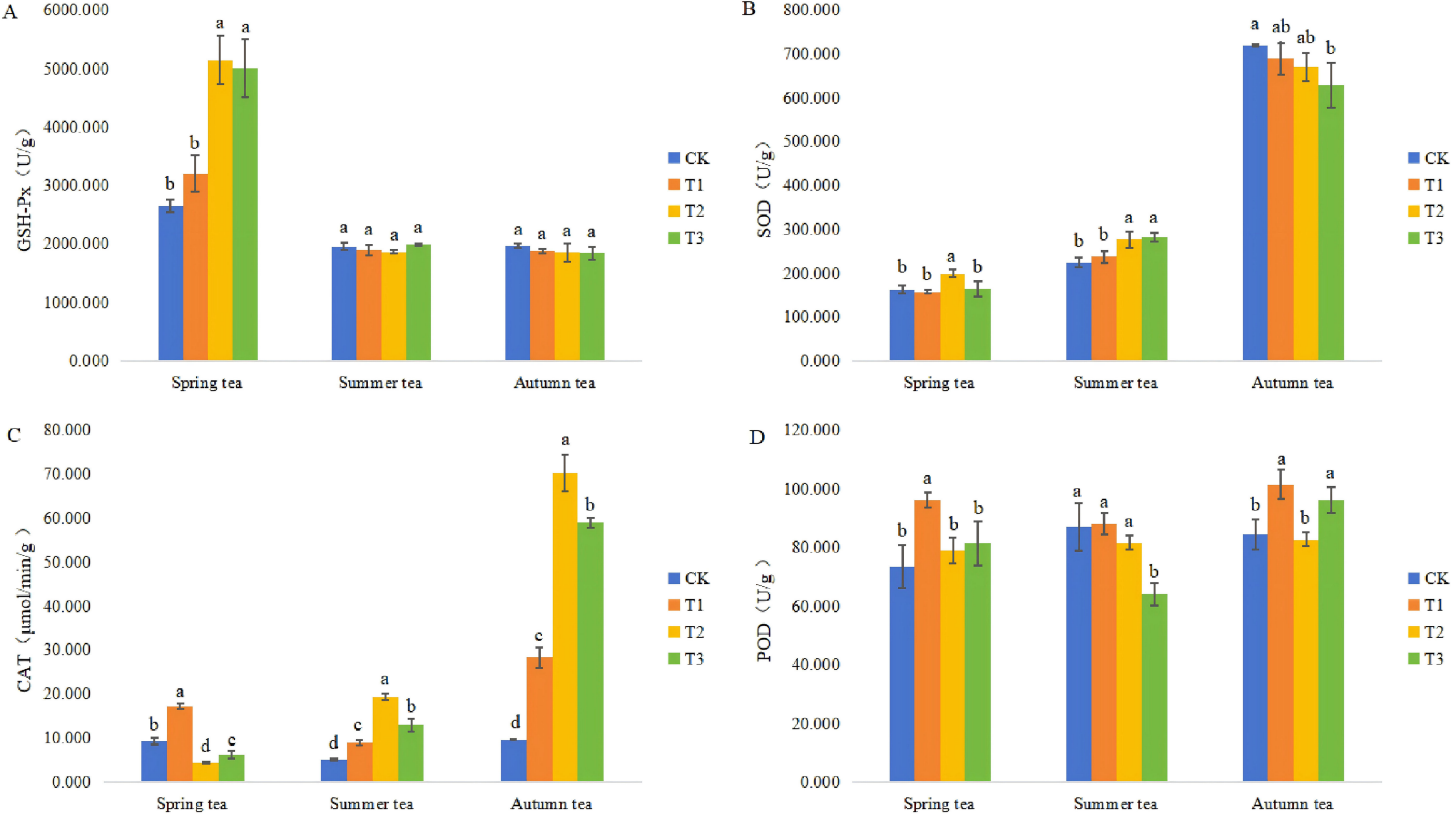
Effect of various treatments on antioxidant enzyme activity in tea. **(A)** glutathione peroxidase; **(B)** superoxide dismutase; **(C)** catalase; **(D)** peroxidase. Different lowercase letters indicate significant differences among treatments in the same season (P<0.05).

The activities of four antioxidant enzymes, glutathione peroxidase (GSH-Px), superoxide dismutase (SOD), catalase (CAT), and peroxidase (POD), exhibited distinct seasonal dynamics and treatment-dependent responses, reflecting the interplay between exogenous Se application, climatic conditions, and plant antioxidant defense strategies (**Figure 4**; **Supplementary Table 5**).

GSH-Px activity showed a pronounced treatment effect exclusively in spring, with T2 and T3 achieving the highest values (5147.1 and 5013.0 U/g), representing 1.94- and 1.89-fold increases over CK (2648.4 U/g) (p < 0.05). By summer and autumn, treatment differences were entirely abolished, with all groups converging to comparable levels (1843-1990 U/g). This seasonal collapse temporally coincided with leaching-driven soil Se decline under high summer precipitation (234.1 mm), most markedly under T2 (-32.6%). Since GSH-Px is a selenocysteine-containing enzyme directly substrate-limited by Se supply, these parallel dynamics mechanistically link soil Se retention to foliar antioxidant capacity, with spring’s low precipitation (82.5 mm) enabling maximal Se uptake and peak GSH-Px activity (**Figure 4A**).

SOD activity increased monotonically across seasons in all treatments (spring < summer < autumn), consistent with progressive ROS accumulation under escalating oxidative stress. T2 recorded the highest spring SOD activity (199.5 U/g, p < 0.05), while T3 became statistically comparable in summer (282.9 U/g). Paradoxically, T3 recorded the lowest autumnal SOD activity (629.5 U/g, p < 0.05) despite maintaining the highest soil available Se and enzyme activities that season. This inverse relationship suggests that robust GSH-Px-mediated ROS scavenging under T3’s superior Se retention partially alleviated cellular H_2_O_2_ burden, reducing demand for SOD-mediated O_2_^-^ dismutation, indicative of functional complementarity among antioxidant systems under Se-sufficient conditions (**Figure 4B**).

CAT activity exhibited the most complex treatment-by-season interaction. In spring, T1 recorded the highest CAT activity (17.24 μmol/min/g), while T2 and T3 showed the lowest values (4.34 and 6.21 μmol/min/g), diametrically opposite to their spring GSH-Px rankings. This reciprocal pattern suggests a metabolic division of labor in H_2_O_2_ detoxification: under abundant Se supply, the efficient selenocysteine-dependent GSH-Px pathway predominates, reducing the catalytic burden on CAT. By autumn, the hierarchy reversed completely, with T2 and T3 exhibiting the highest CAT activities (70.32 and 58.96 μmol/min/g), representing 7.3- and 6.1-fold increases over CK (9.65 μmol/min/g). This autumnal CAT induction is consistent with concurrent SOD peaks, as elevated SOD-mediated O_2_^-^ dismutation generates greater H_2_O_2_ loads requiring enhanced CAT clearance, forming a coordinated SOD to CAT cascade under moderate autumn conditions (24.5°C, 79% humidity) (**Figure 4C**).

**POD** activity remained relatively stable across seasons. T1 consistently maintained the highest POD activity in spring (96.19 U/g) and autumn (101.54 U/g), suggesting selective enhancement of cell wall-associated peroxidative activity and lignification. T3 exhibited the lowest summer POD activity (64.14 U/g), coinciding with its SOD peak, possibly reflecting a resource allocation trade-off favoring primary ROS defense over secondary cell wall reinforcement under maximal oxidative challenge (**Figure 4D**).

Collectively, Se application does not uniformly upregulate all antioxidant enzymes but differentially reconfigures the antioxidant network in a season- and enzyme-specific manner. Spring Se availability predominantly drives GSH-Px-centered enhancement with concomitant CAT suppression, while autumnal conditions trigger a coordinated SOD-CAT cascade amplified by prior Se treatment. These findings underscore the importance of integrating seasonal climatic dynamics into the interpretation of Se-mediated antioxidant responses in tea plants (**Supplementary Table 5**).

### 3.6 The regulation of chlorophyll content in tea by various treatments showed season- and dose-specific profiles

Se treatment effects on chlorophyll were season-specific and dose-selective, with the most prominent protective effect in summer (**Table 3**). Total chlorophyll followed autumn (mean: 0.560 mg/g) > spring (0.482 mg/g) > summer (0.358 mg/g), with summer values declining ~25.7% from spring, consistent with photosynthetic pigment degradation under high temperature and strong light.

**Table 3:** Effect of various treatments on chlorophyll content in tea (mg/g).

Season\Treatments		Chlorophyll content		
		Chlorophyll a	Chlorophyll b	Total Chlorophyll
**Spring**	**CK**	0.347±0.005a	0.114±0.002a	0.461±0.006a
	**T1**	0.353±0.023a	0.120±0.009a	0.473±0.032a
	**T2**	0.369±0.016a	0.126±0.007a	0.496±0.023a
	**T3**	0.371±0.024a	0.127±0.013a	0.498±0.036a
**Summer**	**CK**	0.240±0.021b	0.080±0.008c	0.320±0.029b
	**T1**	0.283±0.003a	0.097±0.002a	0.380±0.005a
	**T2**	0.264±0.002a	0.089±0.000b	0.353±0.002a
	**T3**	0.285±0.002a	0.093±0.001ab	0.378±0.003a
**Autumn**	**CK**	0.405±0.004a	0.143±0.006b	0.548±0.007b
	**T1**	0.409±0.002a	0.144±0.007b	0.553±0.007b
	**T2**	0.413±0.010a	0.157±0.004a	0.570±0.012a
	**T3**	0.415±0.003a	0.153±0.001ab	0.567±0.003a

Different lowercase letters indicate significant differences among treatments in the same season (P<0.05).

In spring, no significant differences in chlorophyll a (0.347-0.371 mg/g), chlorophyll b (0.114-0.127 mg/g), or total chlorophyll (0.461-0.498 mg/g) were observed among treatments (p > 0.05), indicating that vigorous intrinsic synthesis capacity under favorable spring conditions precluded meaningful Se additive effects.

In summer, Se treatments significantly protected chlorophyll against stress-induced degradation. All Se-treated groups maintained significantly higher chlorophyll a than CK (0.240 ± 0.021 mg/g), with T1 (0.283 ± 0.003), T2 (0.264 ± 0.002), and T3 (0.285 ± 0.002 mg/g) not differing among themselves. For chlorophyll b, T1 (0.097 ± 0.002 mg/g) significantly exceeded T2 (0.089 ± 0.000) and CK (0.080 ± 0.008 mg/g), with T3 (0.093 ± 0.001 mg/g) intermediate, demonstrating superior protective efficiency of low-dose Se for chlorophyll b. Total chlorophyll increases over CK reached 18.8% (T1), 18.1% (T3), and 10.0% (T2), all significant (p < 0.05).

In autumn, total chlorophyll rebounded to its annual peak, with treatment effects concentrated in chlorophyll b. Chlorophyll a showed no significant treatment differences. T2 chlorophyll b (0.157 ± 0.004 mg/g) significantly exceeded CK (0.143 ± 0.006) and T1 (0.144 ± 0.007 mg/g), with T3 (0.153 ± 0.001 mg/g) intermediate. Correspondingly, total chlorophyll in T2 (0.570 ± 0.012) and T3 (0.567 ± 0.003 mg/g) significantly exceeded CK (0.548 ± 0.007) and T1 (0.553 ± 0.007 mg/g), indicating that meaningful autumn chlorophyll enhancement requires Se supply ≥ T2 dose.

Across all seasons, chlorophyll b consistently showed greater sensitivity to Se treatments than chlorophyll a, likely reflecting its predominant localization in the light-harvesting complex (LHC) and heightened susceptibility to ROS-mediated degradation under oxidative stress. This pattern suggests that Se-mediated protection of the light-harvesting system represents a key mechanism by which Se promotes photosynthesis in tea plants.

### 3.7 Se treatments exerted season-specific, dose-selective effects on biochemical quality of tea

**Figure 5:**
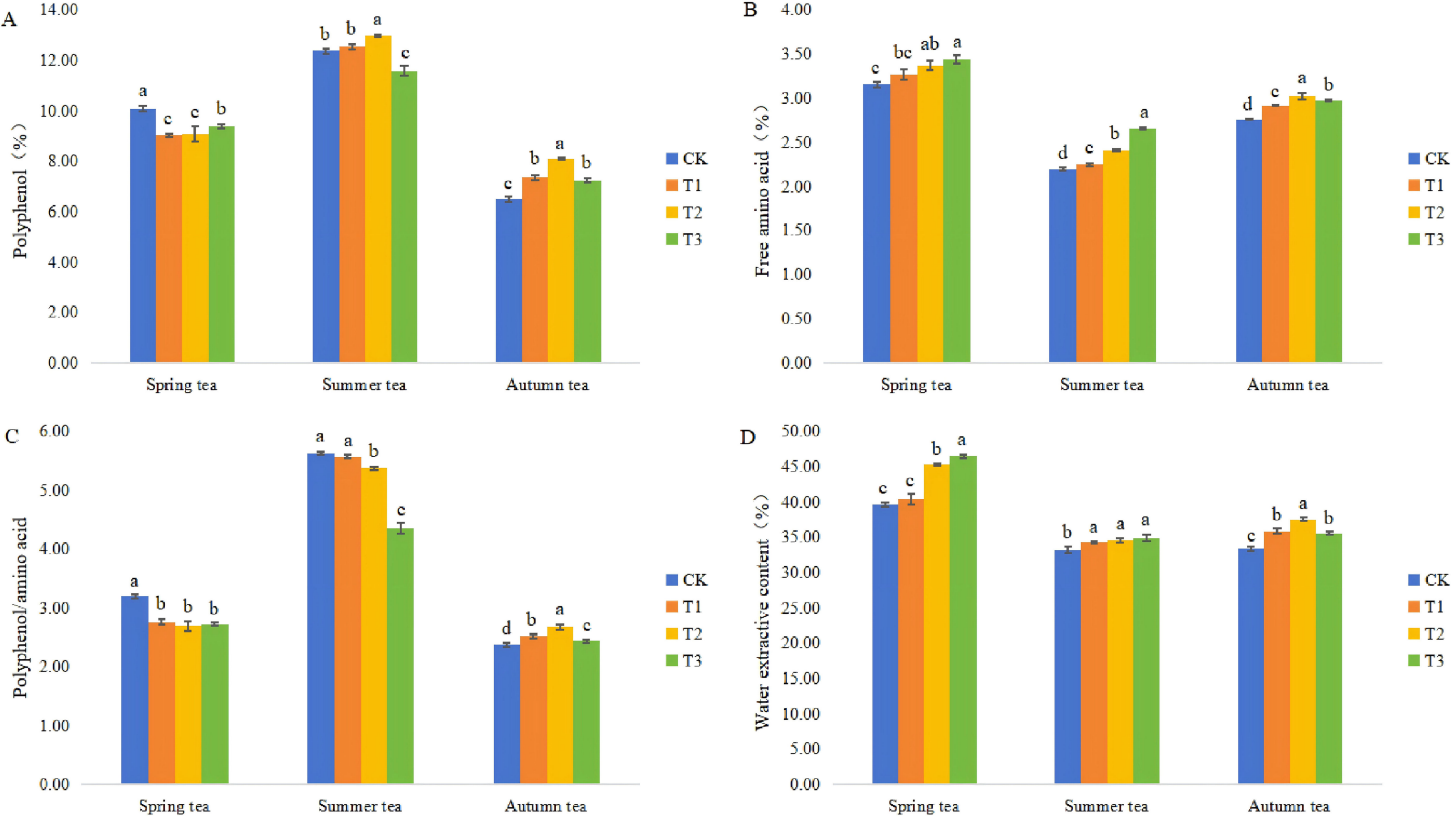
Effect of various treatments on polyphenol and free amino acid content in tea. **(A)** Polyphenol content; **(B)** Free amino acid content; **(C)** polyphenol-to-amino acid ratio; **(D)** water extractive content. different lowercase letters indicate significant differences among treatments in the same season (P<0.05).

**Polyphenol** content followed summer (CK: 12.37 ± 0.10%) > spring (CK: 10.09 ± 0.10%) > autumn (CK: 6.51 ± 0.11%). Se effects were markedly season-dependent in both direction and magnitude. In spring, all Se treatments significantly reduced polyphenol content (T1: −10.5%, T2: −10.0%, T3: −7.0%; p < 0.05), with T1 and T2 not differing significantly. In summer, the response was non-monotonic, T2 produced the highest content (12.97 ± 0.04%, +4.9% over CK), while T3 significantly suppressed it to 11.58 ± 0.19% (−6.4%). In autumn, polyphenols increased progressively with Se dose, peaking under T2 (8.10 ± 0.04%, +24.4% over CK), though T3 (7.25 ± 0.10%) did not differ significantly from T1 (7.35 ± 0.12%) (**Supplementary Table 6**; **Figure 5A**).

Free amino acid content followed the inverse pattern, spring (CK: 3.15 ± 0.03%) > autumn (CK: 2.76 ± 0.01%) > summer (CK: 2.20 ± 0.01%). Unlike polyphenols, Se consistently increased free amino acid content across all seasons, with dose optimum and magnitude varying by season. In spring, content increased with dose to a plateau at T2-T3, with T3 reaching 3.44 ± 0.05% (+9.2%). In summer, a strict dose-dependent response was observed across all treatment levels (p < 0.05), with T3 achieving the largest proportional gain of any season (+20.9%; 2.66 ± 0.01%). In autumn, T2 represented the optimal dose (3.03 ± 0.04%, +9.8%), with T3 (2.98 ± 0.02%) yielding marginal additional benefit (**Supplementary Table 6**; **Figure 5B**).

Polyphenol-to-Amino Acid (P/A) ratio was highest in summer (CK: 5.63 ± 0.03) and lowest in autumn (CK: 2.36 ± 0.04), with spring intermediate (CK: 3.20 ± 0.04). Se application reduced the PA ratio in spring and summer, improving flavor quality, but paradoxically increased it in autumn. In spring, all Se treatments produced equivalent, significant reductions (T1: −13.8%, T2: −15.9%, T3: −14.7%; p < 0.05), indicating that even low-dose Se sufficiently optimizes the spring PA ratio. In summer, T3 produced the largest improvement across all seasons and treatments, reducing the PA ratio from 5.63 ± 0.03 to 4.36 ± 0.09 (−22.6%); T2 yielded a smaller but significant reduction (−4.6%), while T1 had no significant effect. In autumn, the PA ratio increased under all Se treatments, peaking at 2.68 ± 0.04 under T2 (+13.6%), attributable to the disproportionately greater Se-driven enhancement of polyphenols (+24.4%) relative to amino acids (+9.8%). Despite this directional reversal, absolute autumn PA ratio values across all treatments remained within the range characteristic of high-quality green tea (**Supplementary Table 6**; **Figure 5C**).

Water extractive content, a composite indicator of total soluble components governing tea liquor richness, increased with Se across all seasons. In spring, baseline CK was high (39.68 ± 0.32%), already meeting the high-quality green tea criterion (≥36%). Se addition yielded a dose-dependent enhancement: T1 + 1.9% (40.45 ± 0.78%), while T2 + 14.2% (45.33 ± 0.18%) and T3 + 17.1% (46.46 ± 0.26%) were each significantly higher than CK and T1 (p < 0.05), with T3 the highest overall. In summer, CK was lowest (33.20 ± 0.44%), near the threshold (≥32%). All Se treatments raised water extractive content into the first-grade range (≥34%): T1 + 3.4%, T2 + 4.2%, T3 + 5.2% (all p < 0.05); however, differences among Se doses were not significant, indicating a threshold effect. In autumn, Se effects were significant but non-monotonic: T2 achieved the seasonal maximum (37.53 ± 0.21%, +12.6% vs CK 33.32 ± 0.31%), surpassing the ≥36% high-quality threshold and differing from all other treatments (p < 0.05). T1 and T3 were not significantly different (35.88 ± 0.34%, +7.7%; 35.56 ± 0.21%, +6.7%). Overall, T2 emerged as the autumn dose optimum with diminishing returns for higher Se doses (**Supplementary Table 6**; **Figure 5D**).

Taken together, Se application improved all four quality-related indicators in a season-specific manner. In spring, T2 and T3 collectively reduced the PA ratio by ~15% and increased water extractive content by >14% relative to CK, while simultaneously elevating free amino acid content, indicating broad enhancement of spring tea flavor quality. In summer, T3 most effectively addressed the characteristic quality deficiencies of summer tea, markedly reducing the PA ratio (−22.6%) and substantially raising free amino acid content (+20.9%), accompanied by a modest but significant increase in water extractive content (+5.2%). In autumn, T2 emerged as the optimal treatment, simultaneously maximizing water extractive content (+12.6%) and free amino acid content (+9.8%), despite a concurrent PA ratio increase attributable to disproportionately greater polyphenol accumulation. Collectively, these results indicate that Se application enhances the overall biochemical quality profile of tea across all three growth seasons, with T3 most advantageous for summer tea and T2 optimal for autumn tea quality improvement.

## 4 Discussion

This study provides a comprehensive, season-resolved evaluation of Se-enriched organic fertilizer in a perennial tea production system, revealing that Se biofortification is governed by strong interactions among application rate, seasonal environmental conditions, soil biochemical processes, and plant physiological responses. By integrating soil chemistry, enzyme activity, plant Se accumulation, antioxidant systems, photosynthetic traits, and quality-related metabolites across three harvest seasons, our results substantially extend previous single-time-point studies and offer a mechanistic framework for optimizing Se biofortification in tea.

### 4.1 Soil acidification alleviation and seasonal modulation of Se bioavailability

The consistently low soil pH (3.83-4.15) confirms the strongly acidic nature of tea plantation soils in Guangxi, which is known to limit Se bioavailability through adsorption of selenite onto Fe/Al oxides (Liao et al., 2024a). Se-enriched organic fertilizer raises soil pH, most notably in spring under the T2 treatment, suggesting that the organic matrix buffers acidity while supplying Se. This aligns with previous findings that organic amendments can increase soil pH and reduce Al toxicity in tea systems (Jaiswal et al., 2018; Huang et al., 2023).

Organic amendments can also influence Se speciation and sorption-desorption dynamics, linking slower Se release to sustained uptake (Liao et al., 2024a). However, the seasonal attenuation of pH effects (non-significant in autumn) indicates that buffering capacity is transient and likely mediated by microbial decomposition dynamics and rainfall-driven leaching. The summer “threshold effect,” in which all Se treatments produced similar pH increases, further suggests that once a minimal organic input level is reached, additional Se does not proportionally enhance soil chemical amelioration. This has practical implications: moderate application rates may suffice for soil pH regulation, avoiding unnecessary input costs.

The seasonal decline in soil available Se from spring to autumn further highlights the dominant role of environmental processes in governing Se persistence. Although all Se treatments elevated available Se relative to CK, progressive seasonal depletion was observed across all treatments, likely reflecting cumulative plant uptake, microbial immobilization, leaching, and redox-driven transformation into less available forms (Liao et al., 2024b; Qu et al., 2023). The strong precipitation during summer likely accelerated Se migration to deeper soil layers, particularly in the highly weathered red soils used for tea cultivation (Blazina et al., 2014; Liao et al., 2024a). Notably, T3 retained significantly higher available Se throughout the entire season and exhibited the smallest spring-to-autumn decline, suggesting that higher-dose Se-enriched organic fertilizer established a more stable rhizosphere Se reservoir. This sustained Se availability likely reflects gradual mineralization of organically complexed Se and stronger adsorption to organic colloids, reducing immediate losses. By contrast, T1 failed to maintain significant Se enrichment by autumn, indicating that low application rates are insufficient for long-term Se retention under heavy-rainfall conditions.

### 4.2 Enzyme-specific and season-dependent restructuring of soil biochemical functioning

Soil enzyme activities provided important mechanistic insight into how Se altered rhizosphere biochemical functioning. Soil enzyme responses revealed highly dynamic and non-linear regulation, underscoring that Se acts as both a micronutrient and a stressor depending on dose and environmental context. The pronounced season × treatment interactions observed for all four enzymes indicate that microbial community responses to Se are tightly coupled to temperature, moisture, and rhizosphere activity (Wang et al., 2022; Qu et al., 2023), while soil OM quality may alter substrate availability, influencing enzyme kinetics and responses to Se (Jia et al., 2025; He et al., 2026).

Catalase activity, which reflects microbial oxidative stress responses and peroxide detoxification capacity (Revina et al., 2024; Daunoras et al., 2024), exhibited pronounced seasonal shifts in optimal treatment, with T2 maximizing activity in summer and T3 in autumn. This suggests that moderate Se may alleviate oxidative stress under peak summer heat, whereas higher Se concentrations become advantageous under cooler autumn conditions. The spring suppression of catalase under T2 may indicate reduced ROS burden due to lower environmental stress. Similar biphasic responses have been reported in soil microbial communities exposed to trace Se, where low concentrations stimulate antioxidant metabolism but higher concentrations inhibit enzyme activity through protein interaction or microbial community restructuring (Liao et al., 2024a; Somagattu et al., 2024; Fang et al., 2026).

Urease exhibited the most dramatic seasonal reversal, especially under T2, where activity declined sharply in summer before rebounding to the highest level in autumn. Since urease mediates nitrogen mineralization through urea hydrolysis, these dynamics suggest strong Se regulation of microbial nitrogen cycling (Jia et al., 2025). High summer rainfall may have disrupted ureolytic microbial populations or reduced substrate accessibility through waterlogging-induced oxygen limitation (Gu et al., 2019). However, the strong autumn recovery under T2 suggests that once climatic stress declined, Se-enhanced microbial communities resumed highly active nitrogen turnover. T3 displayed comparatively stable urease activity across seasons, indicating that higher Se supply may confer greater microbial resilience.

The consistent suppression of invertase in summer across all Se treatments indicates a robust inhibitory effect on carbon cycling under high-temperature conditions (Hursh et al., 2017; Jiang et al., 2021; Zhang et al., 2024). This may reflect a shift in microbial resource allocation away from labile carbon metabolism under combined Se and heat stress (Ren et al., 2024). In contrast, the uniform stimulation of acid phosphatase in summer suggests enhanced phosphorus mobilization, potentially compensating for increased plant demand or reduced P availability under stress conditions (Zuo et al., 2021).

Collectively, these results demonstrate that Se-enriched organic fertilizer does not exert uniform effects on soil biochemical functioning; rather, it restructures microbial processes in a season-specific manner. This highlights the importance of considering temporal dynamics when evaluating soil amendments in perennial systems.

### 4.3 Decoupling and coupling of soil-plant Se dynamics across seasons

A key finding of this study is the shifting relationship between soil available Se and tea leaf Se content across seasons. In spring, despite significant differences in soil Se, leaf Se concentrations plateaued across T1-T3, indicating a saturation of uptake capacity. This suggests that under favorable spring conditions, tea plants rapidly reach a physiological ceiling for Se assimilation, likely constrained by transporter capacity, assimilation enzymes, or vacuolar storage limits. This is consistent with previous reports that plant Se uptake is regulated by transporter activity and metabolic constraints rather than solely by external availability (Song et al., 2018; Liao et al., 2024b). The lack of further leaf enrichment despite higher soil Se strongly supports the existence of such saturation mechanisms.

In contrast, the strong dose-dependent relationship observed in summer indicates that Se uptake becomes supply-limited under stress conditions. High temperature, intense radiation, and elevated evapotranspiration likely reduced root metabolic efficiency and membrane transport capacity, making external Se concentration the dominant determinant of uptake (Qu et al., 2023; Liao et al., 2024a).

The autumn results reveal a more complex scenario. Despite the convergence of soil Se between T1 and CK, leaf Se remained significantly elevated under T1, suggesting alternative uptake mechanisms such as deeper root absorption or remobilization from internal pools. This decoupling highlights the importance of considering plant physiological adaptations, including root system architecture and internal Se redistribution, in biofortification strategies (Qu et al., 2023).

Notably, T2 and T3 treatments not only maintained but enhanced autumn Se accumulation beyond spring levels, indicating that higher Se inputs can overcome seasonal limitations and sustain enrichment. This is particularly important given that autumn tea contributes significantly to annual production in some regions.

### 4.4 Antioxidant system reprogramming under Se supply: evidence for compensatory regulation

The antioxidant enzyme data reveal a coordinated but highly plastic response to Se, reflecting its dual role as an essential micronutrient and a modulator of oxidative stress (Ikram et al., 2024). Spring showed the clearest Se-driven activation of glutathione peroxidase (GSH-Px), with T2 and T3 nearly doubling activity relative to CK. Because GSH-Px is a selenium-dependent enzyme containing selenocysteine at its catalytic center, this response provides strong evidence that increased Se uptake directly enhanced selenoprotein synthesis or activity (Zhang et al., 2020). However, the disappearance of treatment effects in summer and autumn suggests that environmental stress overrides Se-mediated regulation, leading to a convergence of GSH-Px activity. This indicates that Se supplementation alone may not fully compensate for environmental constraints on antioxidant systems, integrating soil OM management and moisture control can augment redox buffering.

The reciprocal behavior of GSH-Px and catalase (CAT) in spring is particularly noteworthy. High Se doses strongly activated GSH-Px while simultaneously suppressing CAT, suggesting functional compensation between H_2_O_2_ detoxification pathways. Under Se sufficiency, the highly efficient glutathione-dependent pathway may reduce peroxide levels sufficiently to decrease reliance on CAT. By autumn, this relationship reversed completely, with CAT becoming strongly induced under T2 and T3.

The observed trade-off between SOD and CAT activities in autumn, where high Se suppressed SOD but strongly enhanced CAT, provides evidence for compensatory regulation within the antioxidant network (Ikram et al., 2024). Such shifts may reflect optimization of reactive oxygen species (ROS) detoxification pathways, where increased CAT activity reduces hydrogen peroxide accumulation, thereby reducing the need for upstream SOD activity.

Similarly, the differential regulation of POD across seasons and treatments further supports the notion of a flexible, Se-modulated antioxidant system (Ikram et al., 2024). These findings align with previous studies showing that Se can reprogram antioxidant metabolism through both enzymatic and non-enzymatic pathways (Gorni et al., 2025).

### 4.5 Protection of photosynthetic apparatus under stress: selective sensitivity of chlorophyll b

The chlorophyll results indicate that Se-mediated protection of photosynthesis is particularly important during summer stress. Chlorophyll degradation under high temperature and strong irradiance is a well-established consequence of oxidative damage to chloroplast membranes and pigment-protein complexes (Ashraf and Harris, 2013; Gururani et al., 2015; Shi et al., 2022). The approximately 26% decline in summer total chlorophyll in CK reflects severe photosynthetic stress. Se treatment significantly alleviated this loss, confirming that Se improved chloroplast stability and reduced oxidative pigment degradation. The greater sensitivity of chlorophyll b compared to chlorophyll a suggests that Se primarily stabilizes the light-harvesting complex (LHC), which is more vulnerable to oxidative damage (Lanza and Reis, 2021). This suggests that Se preferentially protected antenna complexes rather than reaction centers.

The superior performance of T1 for chlorophyll b protection indicates that low Se doses may be sufficient, or even optimal, for preserving photosynthetic efficiency under stress. This is consistent with the concept of hormesis, where low levels of a stressor elicit beneficial responses, while higher levels may be less effective or even inhibitory (Yin et al., 2019).

The lack of significant effects in spring further supports the idea that Se benefits are most pronounced under stress conditions, rather than under optimal growth environments.

### 4.6 Integrated effects on tea quality: balancing nutrition and sensory attributes

Se application significantly improved key quality parameters, but the effects were highly season-dependent. The consistent increase in free amino acids across all seasons is particularly important, as these compounds contribute to umami flavor and are positively associated with tea quality (Liao et al., 2025).

The reduction of polyphenols in spring and the consequent decrease in the polyphenol-to-amino acid (P/A) ratio indicate improved flavor balance, reducing astringency. In summer, the strong reduction in P/A ratio under T3 suggests that high-dose Se can effectively mitigate the typically poor quality of summer tea, characterized by high bitterness and low amino acid content.

In autumn, the increase in both polyphenols and amino acids under T2 resulted in a higher P/A ratio, yet values remained within the acceptable range for high-quality tea. This suggests that Se can enhance overall biochemical richness without compromising sensory acceptability.

The consistent increase in water extractives across all seasons further indicates improved infusion quality, reflecting enhanced accumulation of soluble compounds. The identification of T2 as the optimal autumn treatment and T3 as the optimal summer treatment underscores the necessity of season-specific management strategies.

## 5 Conclusions

This study provides the integrated, seasonally resolved analysis of Se-enriched organic fertilizer effects in a tea plantation system. The findings reveal strong season-dependent interactions governing soil Se dynamics, enzyme activity, plant uptake, antioxidant responses, and quality traits. By demonstrating that optimal Se application rates vary across seasons, this work advances the development of precision biofortification strategies that enhance both nutritional value and sensory quality of tea, while maintaining soil health and sustainability. Future research should elucidate the molecular mechanisms of Se uptake and metabolism in tea under varying environmental conditions, and evaluate the long-term ecological impacts of repeated Se application.

## Data Availability

The original contributions presented in the study are included in the article/**Supplementary Material**. Further inquiries can be directed to the corresponding author.
