# Supplementary material for: Seasonal regulation of selenium biofortification in tea through Se-rich organic fertilizer application
Source: Front Plant Sci. 2026 Jul 15;17:1883624. doi: 10.3389/fpls.2026.1883624 (PMC13415369; doi:10.3389/fpls.2026.1883624)
Supplement: Supplementary file 1 [file Table1.docx]

**Table S1 Meteorological information recorded during the three sampling months**

|  | **April 2025** | **July 2025** | **October 2025** |
| --- | --- | --- | --- |
| **Temperature range (℃)** | 11.4-35.9 | 24.1-34.1 | 14.9-31.5 |
| **Average temperature (℃)** | 24.5 | 28.9 | 24.5 |
| **Precipitation (**mm) | 82.5 | 234.1 | 105.4 |
| **Humidity (%)** | 70 | 86 | 79 |
| **Total illumination (hr)** | 235.6 | 283.3 | 278.4 |

**Table S2 Effect of various treatments on soil enzyme activities**

| **Enzyme** | **Treatments** | **Spring** | **Summer** | **Autumn** |
| --- | --- | --- | --- | --- |
| **Catalase**  **(U/g)** | **CK** | 718.53±19.50a | 818.53±9.19c | 701.30±5.71b |
|  | **T1** | 693.37±27.77a | 847.17±3.46b | 682.33±6.83b |
|  | **T2** | 497.23±50.80b | 1013.60±11.32a | 646.30±29.55c |
|  | **T3** | 698.13±20.17a | 579.53±19.21d | 1003.53±10.10a |
| **Urease**  **(U/g)** | **CK** | 7649.27±218.56b | 7197.63±168.81b | 3365.77±186.99d |
|  | **T1** | 8101.53±289.26a | 7724.17±66.10a | 6812.13±134.52c |
|  | **T2** | 6287.73±240.41c | 3216.47±50.26c | 9247.97±188.00a |
|  | **T3** | 8292.23±243.39a | 7077.80±295.30a | 8445.17±355.42b |
| **Invertase**  **(U/g)** | **CK** | 313.53±4.97b | 344.77±0.87a | 327.40±3.02b |
|  | **T1** | 357.87±14.50a | 235.83±11.31b | 372.57±8.35a |
|  | **T2** | 330.53±14.15b | 214.67±12.37c | 288.93±12.39c |
|  | **T3** | 194.57±3.06c | 186.77±6.86d | 379.67±4.02a |
| **Acid Phosphatase**  **(IU/g)** | **CK** | 0.0810±0.0019ab | 0.0604±0.0017c | 0.0701±0.0049b |
|  | **T1** | 0.0818±0.0008a | 0.0790±0.0007a | 0.0712±0.0197b |
|  | **T2** | 0.0783±0.0020b | 0.0730±0.0013b | 0.0553±0.0007c |
|  | **T3** | 0.0511±0.0008c | 0.0810±0.0005a | 0.0990±0.0029a |

Note: Different lowercase letters within the same column indicate significant differences among treatments in the same season (P<0.05).

**Table S3 Effect of various treatments on soil available Se content (mg/kg)**

| **Treatments** | **Spring** | **Summer** | **Autumn** |
| --- | --- | --- | --- |
| **CK** | 0.0351±0.0031d | 0.0357±0.0008d | 0.0203±0.0021c |
| **T1** | 0.0435±0.0019c | 0.0413±0.0017c | 0.0237±0.0002c |
| **T2** | 0.0709±0.0024b | 0.0478±0.0021b | 0.0331±0.0012b |
| **T3** | 0.0788±0.0009a | 0.0589±0.0018a | 0.0559±0.0022a |

Note: Different lowercase letters indicate significant differences among treatments in the same season (P<0.05).

**Table S4 Effect of various treatments on Se content in tea (mg/kg)**

| **Treatments** | **Spring tea** | **Summer tea** | **Autumn tea** |
| --- | --- | --- | --- |
| **CK** | 0.1970±0.0164b | 0.1199±0.0067c | 0.1465±0.0048c |
| **T1** | 0.5998±0.0204a | 0.3127±0.0079b | 0.5840±0.0123b |
| **T2** | 0.6240±0.0385a | 0.4590±0.0510a | 0.6660±0.0554a |
| **T3** | 0.6491±0.0537a | 0.5105±0.0079a | 0.6572±0.0262a |

Note: Different lowercase letters indicate significant differences among treatments in the same season (P<0.05).

**Table S5 Effect of various treatments on antioxidant enzyme activity in tea**

| **Enzyme** | **Treatments** | **Spring tea** | **Summer tea** | **Autumn tea** |
| --- | --- | --- | --- | --- |
| **GSH-Px**  **(U/g)** | CK | 2648.408±111.966b | 1960.155±65.869a | 1967.984±41.610a |
|  | T1 | 3200.679±310.408b | 1893.323±93.997a | 1887.342±35.717a |
|  | T2 | 5147.05±407.986a | 1865.103±29.834a | 1852.698±158.726a |
|  | T3 | 5013.03±495.680a | 1990.512±22.261a | 1843.053±115.558a |
| **SOD**  **(U/g)** | CK | 163.245±8.509b | 224.404±11.377b | 720.158±3.124a |
|  | T1 | 157.875±3.512b | 237.236±13.559b | 689.317±36.004ab |
|  | T2 | 199.494±9.067a | 276.618±19.132a | 671.178±31.962ab |
|  | T3 | 163.927±17.107b | 282.927±10.641a | 629.525±51.715b |
| **CAT**  **(μmol/min/g)** | CK | 9.311±0.726b | 5.071±0.240d | 9.653±0.157d |
|  | T1 | 17.240±0.688a | 8.952±0.601c | 28.325±2.294c |
|  | T2 | 4.335±0.217d | 19.361±0.631a | 70.321±4.217a |
|  | T3 | 6.206±0.850c | 13.044±1.415b | 58.958±1.153b |
| **POD**  **(U/g)** | CK | 73.421±7.444b | 87.146±8.108a | 84.613±5.129b |
|  | T1 | 96.190±2.540a | 88.285±3.678a | 101.542±4.848a |
|  | T2 | 79.061±4.505b | 81.676±2.279a | 82.784±2.272b |
|  | T3 | 81.381±7.640b | 64.139±3.770b | 96.264±4.573a |

Note: Different lowercase letters indicate significant differences among treatments in the same season (P<0.05).

**Table S6 Effect of various treatments on the biochemical quality of tea**

|  | | **polyphenol (%)** | **free amino acid (%)** | **Polyphenol/amino acid** | **Water extractive** |
| --- | --- | --- | --- | --- | --- |
| **Spring tea** | **CK** | 10.09±0.10a | 3.15±0.03c | 3.20±0.04a | 39.68±0.32c |
|  | **T1** | 9.03±0.06c | 3.27±0.07bc | 2.76±0.04b | 40.45±0.78c |
|  | **T2** | 9.08±0.31c | 3.37±0.05ab | 2.69±0.08b | 45.33±0.18b |
|  | **T3** | 9.38±0.08b | 3.44±0.05a | 2.73±0.03b | 46.46±0.26a |
| **Summer tea** | **CK** | 12.37±0.10b | 2.20±0.01d | 5.63±0.03a | 33.20±0.44b |
|  | **T1** | 12.55±0.11b | 2.25±0.02c | 5.57±0.02a | 34.33±0.13a |
|  | **T2** | 12.97±0.04a | 2.41±0.01b | 5.37±0.03b | 34.60±0.32a |
|  | **T3** | 11.58±0.19c | 2.66±0.01a | 4.36±0.09c | 34.94±0.45a |
| **Autumn tea** | **CK** | 6.51±0.11c | 2.76±0.01d | 2.36±0.04d | 33.32±0.31c |
|  | **T1** | 7.35±0.12b | 2.92±0.01c | 2.52±0.04b | 35.88±0.34b |
|  | **T2** | 8.10±0.04a | 3.03±0.04a | 2.68±0.04a | 37.53±0.21a |
|  | **T3** | 7.25±0.10b | 2.98±0.02b | 2.44±0.02c | 35.56±0.21b |

Note: Different lowercase letters indicate significant differences among treatments in the same season (P<0.05).
